# Tumor-Derived Suppressor of Fused Mutations Reveal Hedgehog Pathway Interactions

**DOI:** 10.1371/journal.pone.0168031

**Published:** 2016-12-28

**Authors:** Nicole M. Urman, Amar Mirza, Scott X. Atwood, Ramon J. Whitson, Kavita Y. Sarin, Jean Y. Tang, Anthony E. Oro

**Affiliations:** 1 Program in Epithelial Biology and Department of Dermatology, Stanford University School of Medicine, Stanford, CA, United States of America; 2 Department of Developmental and Cell Biology, Chao Family Comprehensive Cancer Center, University of California, Irvine, Irvine, CA, United States of America; National Cancer Center, JAPAN

## Abstract

The Hedgehog pathway is a potent regulator of cellular growth and plays a central role in the development of many cancers including basal cell carcinoma (BCC). The majority of BCCs arise from mutations in the Patched receptor resulting in constitutive activation of the Hedgehog pathway. Secondary driver mutations promote BCC oncogenesis and occur frequently due to the high mutational burden resulting from sun exposure of the skin. Here, we uncover novel secondary mutations in Suppressor of Fused (SUFU), the major negative regulator of the Hedgehog pathway. SUFU normally binds to a Hedgehog transcriptional activator, GLI1, in order to prevent it from initiating transcription of Hedgehog target genes. We sequenced tumor-normal pairs from patients with early sporadic BCCs. This resulted in the discovery of nine mutations in SUFU, which were functionally investigated to determine whether they help drive BCC formation. Our results show that four of the SUFU mutations inappropriately activate the Hedgehog pathway, suggesting they may act as driver mutations for BCC development. Indeed, all four of the loss of function SUFU variants were found to disrupt its binding to GLI, leading to constitutive pathway activation. Our results from functional characterization of these mutations shed light on SUFU’s role in Hedgehog signaling, tumor progression, and highlight a way in which BCCs can arise.

## Introduction

The Hedgehog (HH) pathway drives downstream proliferative and maintenance signaling programs in many developmental and tissue homeostasis processes and has been shown to play a pivotal role in the development of many cancers [1]. Hedgehog pathway activation occurs upon binding of HH ligand to the receptor Patched1 (PTCH1) on the cell’s primary cilium, causing de-repression of the G-protein coupled receptor Smoothened (SMO) and resultant activation of Glioma-associated homologue (GLI) transcription factors. In vertebrates, Suppressor of Fused (SUFU) serves as a key major negative HH pathway regulator, the loss of which results in ectopic high-level pathway activity [2]. SUFU suppresses GLI1 activity through direct binding to its N- and C-domains, sequestering it in the cytoplasm, and scaffolding GLI1 to transcriptional co-repressors[3,4]. SMO activation uncouples SUFU and activates GLI1, with loss of SUFU phenocopying mutations in PTCH1[2,5]. Conceivably, any alteration in SUFU/GLI1 interactions could result in over-proliferation and cell malignancy.

Basal cell carcinoma (BCC), a cancer of epidermal basal keratinocytes, is the most common cancer in the United States. BCCs are locally invasive epithelial tumors that are caused by activating mutations in the HH pathway, typically through the loss of the receptor PTCH1 or by activating SMO. BCCs represent an ideal model system to study mechanisms of cancer development in HH-driven tumors due to their exceptionally high mutational load from exposure to environmental mutagens and their ease of accessibility [6]. Genomic analysis has revealed that BCCs are typically diploid and carry a high frequency of non-silent single nucleotide variants compared to other tumor types[6–8]. Indeed, BCCs bear the highest rate of recurrent mutations in all cancers at 65 mutations per megabase[8]. PTCH1 and SMO mutations have been described in BCC development, but missense mutations in other HH proteins like SUFU are also frequently found and are of unknown significance. Recent studies reveal that up to 8% of BCCs possess SUFU variants [8], our previous analysis indicates that up to 50% of BCC variants are functionally silent and requiring functional validation (6). How clinically observed SUFU variants alter SUFU function remains poorly described.

Here, we uncover and functionally validate nine SUFU mutations from sequencing 58 sporadic human BCC tumor-normal pairs from patients with and without Basal Cell Nevus Syndrome (Gorlin’s Syndrome). We further interrogated these clinically derived variants to describe functional significance and determine whether they had the potential to drive repressor-inactivating pathways for BCC formation. Validation of HH mutations provides valuable information on which variants serve as drivers of suppressors of cancer progression and help tailor personalized therapy for Hedgehog-driven cancers.

## Materials and Methods

### Case Samples

After Stanford Human Subjects panel approval (IRB protocol #29381), written informed consent was obtained from patients 18 years or older for tumor sequencing. 15 sporadic BCCs and 43 BCCs obtained from patients with Basal Cell Nevus Syndrome were sequenced along with normal patient-matched samples. The sequencing data for all mutations except P191S is deposited in the NIH Sequence Read Archive, accession number SRP079235. P191S is deposited in Gene Expression Omnibus, accession number GSE58377, as cited in “Bonilla X, Parmentier L, King B, et. al., Genomic analysis identifies new mutations and progression pathways in skin basal cell carcinoma. Nat Genet. 2016 Apr;48(4):398–406. doi: 10.1038/ng.3525. Epub 2016 Mar 7. PubMed PMID: 26950094.”

### Targeted Re-Sequencing with Fluidigm

58 BCCs, with 43 of these BCCs obtained from patients with Basal Cell Nevus Syndrome, were sequenced along with normal patient-matched samples. Five to eight 10-μm sections were obtained from the formalin-fixed, paraffin-embedded (FFPE) tumor block, and DNA was isolated using the QIAGEN DNeasy blood and tissue kit according to manufacturer’s protocol (QIAGEN). The exonic regions of *SMO* and *SUFU* were amplified using the Access Array platform (Fluidigm). The samples were amplified in a multiplex format with genomic DNA (100 ng) according to the manufacturer’s recommendation (Ambry Genetics). Subsequently, the multiplexed library pools were subjected to deep sequencing using the Illumina MiSeq platform. After demultiplexing and FASTQ file generation for the raw data, 150 base pair reads were aligned to the human reference genome sequence (hg19) using the BWA aligner. Samtools mpileup was used to call variants. Only bases meeting the minimum mapping quality score of 20 were considered. Calls required a minimum allele frequency of 5% at a position with a read depth of >100. Identified variants were annotated using SeattleSeq138 to exclude non-pathogenic variants reported in dbSNP138 and to identify variants that had non-synonymous consequences or affected splice sites.

### Cloning and Cell Culture

Sequence verified SUFU variants were created using the InFusion cloning kit (Clontech) and cloned into peGFP-C1 (Clontech) and pCDH (SBI) vectors. *Sufu*-null MEFs [2] and HEK-293T cells were cultured in DMEM media supplemented with 10% fetal bovine serum. ASZ001 [9] were cultured in 154CF media (Life Technologies) supplemented with 2% chelated fetal bovine serum and 0.05mM CaCl_2_.

For qRT-PCR experiments, each peGFP-C1-SUFU construct was nucleofected into *Sufu*-null MEFs using the Amaxa nucleofection kit and protocol, grown to confluency in DMEM + 10% fetal bovine serum, and then withdrawn from serum in DMEM for 24 hours to ensure each cell was in the G0 phase of the cell cycle. Nucleofection was done with 1 μg of DNA into 100 uL of media containing *Sufu-*null MEF cells and nucleofection reagent. After nucleofection, cells were plated onto a 24-well plate with 1 mL of DMEM supplemented with 10% fetal bovine serum per well. Cells were incubated for 24 hours then RNA was purified for qRT-PCR.

### Quantitative RT-PCR

qRT-PCR was used to validate the activity of each SUFU mutant, under the standard SYBR green protocol in Mx3000P qPCR system (Agilent). The fold change in mRNA expression of the HH target gene *Gli1* was measured using ΔΔCt analysis with *Gapdh* as an internal control gene using primer sequences as previously described (5).

### Statistical Analysis

Each biological replicate for each SUFU variant was compared across trials by standardizing each sample to wild type. Biological replicates for each variant were aggregated and plotted as mean ± SEM. Significance was determined by using an unpaired t test with equal SD, comparing each mutant to wild type. P values were calculated with a two-tailed comparison.

### Immunoprecipitation

HEK-293T cells were co-transfected with equimolar amounts of a tandem-epitope-tagged SUFU variant and GFP-GLI1 using Lipofectamine. After addition of anti-HA-antibody coated magnetic beads (Thermo Fisher Scientific) and a 4° C overnight incubation, a magnetic stand was used to pull down the beads prior to washing. Samples were immunoblotted using primary antibodies against the following proteins: GLI1 (Cell Signaling 2643S), GAPDH (Santa Cruz Biotechnology sc-32233), GFP (Cell Signaling 2555S) and HA (Abcam ab9134) at 1:1000 dilution in 5% BSA. Fluorescent secondary antibodies were used (Licor) and then imaged using an Odyssey scanner (Licor).

### Real Time Glow Luciferase Assay

peGFP-C1 SUFU constructs were nucleofected into ASZs and plated at 50% confluency into a 96-well plate in 6 biological replicates. After 24 hours, cells were treated with 154CF (Life Technologies) supplemented with Real Time Glow reagent per manufacture’s instructions (Promega). Time points were taken every few hours for 2 days to generate growth curves for each SUFU variant. Each well was normalized to itself after a three-hour equilibration to control for plating variation. Luminescence was measured by the SpectraMax M5 plate reader (Molecular Devices) at 37°C over a 1.5 second interval. SUFU overexpression via nucleofection in ASZ001 cells was confirmed via immunoblotting. Primary antibodies against HA (Abcam ab9134) and Lamin A + C (Abcam ab8984) as a loading control were used.

### Nuclear/Cytoplasmic Fractionation

NIH 3T3 cells were transfected with peGFP-C1 SUFU constructs using Lipofectamine LTX supplemented with Plus reagent. After 24 hours, the cells were withdrawn from serum and treated with 30nM Smoothened agonist (SAG) in order to induce HH expression. After 16 hours, nuclei were purified by mechanical disruption in hypotonic lysis buffer (Sigma) followed by pelleting at 800 rcf. The nuclei were then lysed in RIPA buffer supplemented with protease inhibitor and benzonase for 15 minutes. Clarified samples were immunoblotted using primary antibodies against GFP (Cell Signaling 2555S), GLI1 (Cell Signaling 2643S), and Lamin A + C (Abcam ab8984) as a loading control at 1:1000 dilution in 5% BSA. Fluorescent secondary antibodies were used (Licor) and then imaged using an Odyssey scanner (Licor).

## Results

### Targeted Re-Sequencing with Fluidigm Reveals 9 SUFU Mutations

**Fig 1:**
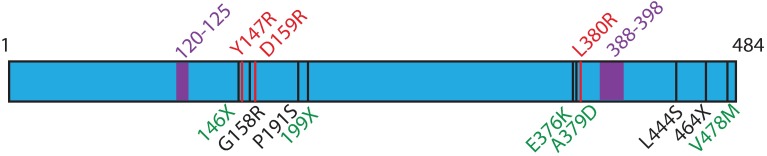
SUFU variants in basal cell carcinoma. Whole exome sequencing with targeted re-sequencing of sporadic and Gorlin’s syndrome tumor-normal pairs revealed 9 variants, depicted in black on the SUFU gene map. Gorlin’s mutations are shown in green, while sporadic mutations are in black. Red variants are previously known SUFU mutations that disrupt SUFU-GLI1 interactions [12]. Highlighted purple regions are GLI1 binding sites. 120–125 binds the C-terminus of GLI1 [12], while 388–398 binds the N-terminus of GLI1[4].

We previously performed whole exome and targeted re-sequencing of 58 sporadic and Gorlin’s Syndrome BCC samples and found expected mutations in PTCH1 and SMO in accordance with our previous publications [6,7]. Interestingly, we also identified 9 SUFU variants, many of which had not been previously described. SUFU is a 484-amino acid long protein, and missense and nonsense mutations were found at residues 146 (Gorlin’s), 158 (Sporadic), 191 (Sporadic), 199 (Gorlin’s), 376 (Gorlin’s), 379 (Gorlin’s), 444 (Sporadic), 464 (Sporadic), and 478 (Gorlin’s) as illustrated in Fig 1. Interestingly, these mutations did not overlap with another study investigating SUFU mutations in multiple meningioma [10]. In addition, by examining both the COSMIC and TCGA databases, only 146X was previously found in 2 cases of esophageal cancer [11] with the remaining mutations not previously described.

### SUFU Variants are Both Passenger and Loss of Function Mutations

**Fig 2:**
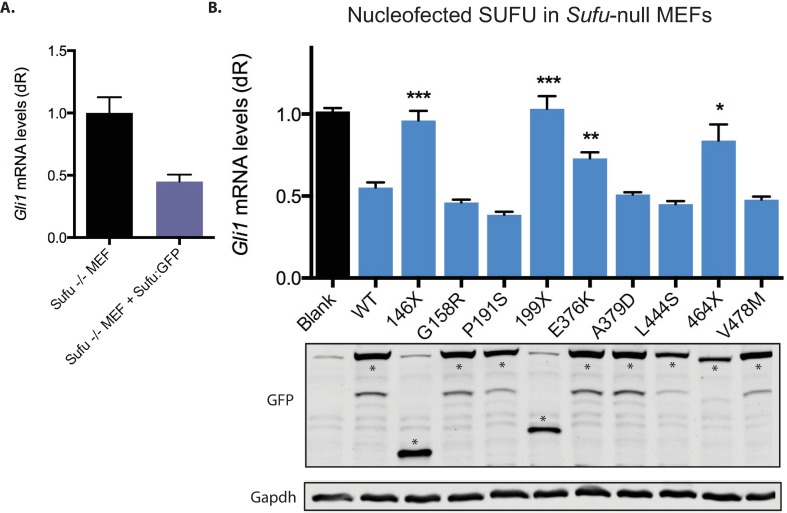
SUFU variants show differential suppression of HH signaling. (A). *Sufu*-null MEFs nucleofected with either GFP or wild type SUFU:GFP, *Gli1* mRNA is used as a readout of Hh pathway activity. (B). qRTPCR used to determine the activity of each SUFU variant, with a Western blot shown directly above to show that each protein is comparably expressed. SUFU:GFP appears as expected at 81 kDa, with truncations 146X and 199X appearing at 48 kDa and 53 kDa, respectively. Gapdh is a loading control. Asterisks denote the correct band on the GFP blot. * is P<0.005, ** is P<0.0005, *** is P<0.0001. Significance was determined by using an unpaired t test with equal SD, comparing each mutant to wild type. P values were calculated with a two-tailed comparison.

To determine how these SUFU mutations alter the HH pathway, we developed a functional assay using *Sufu*-null mouse embryonic fibroblasts (MEFs; Fig 2A). We compared *Sufu*-null MEFs nucleofected with wild type SUFU:GFP or mock nucleofected with GFP. As expected, the mock nucleofected *Sufu*-null MEFs showed a high amount of *Gli1* mRNA—a HH target gene and signaling readout. In *Sufu*-null MEFs with nucleofected wild type SUFU:GFP, we saw *Gli1* mRNA levels reduced by half, validating that SUFU was functioning normally as a negative repressor of the HH pathway. We conclude that the *Sufu*-null MEFs provide a sensitive and convenient test of SUFU function.

We next tested each SUFU variant’s effect on HH signaling in *Sufu*-null MEFs by quantifying their levels of *Gli1* mRNA compared to wild type SUFU. Residues 159 and 380 are known to affect SUFU’s ability to degrade GLI1 [12]. As G158R and A379D were only one amino acid away from these pivotal residues, we hypothesized that perhaps these SUFU variants would also be important for regulating the interaction between SUFU and GLI1. Surprisingly, SUFU variants G158R and A379D displayed wild type levels of suppression, suggesting that these variants are most likely passenger mutations. The SUFU variants that displayed a loss of function compared to wild type included 146X, 199X, E376K, and 464X, while the other five variants demonstrated wild type SUFU activity (Fig 2B). Three of the loss of function variants are nonsense mutations that result in a truncated protein and one is a missense mutation. Examination of the protein by western blotting indicates each has the appropriate size and comparable levels of protein (Fig 2B). We conclude that G158R, P191S, A379D, L444S, and V478M are likely passenger mutations.

### Disruption of SUFU-GLI Binding Reveals a Repressor-Inactivating Mechanism for Basal Cell Carcinoma Growth

SUFU binds and regulates GLI1 through two distinct regions: the carboxy-terminal end of SUFU binds to the amino-terminal end of GLI1, and the amino-terminal end of SUFU binds to the carboxy-terminal tail of GLI1 [4]. Three of the SUFU mutations are nonsense mutations that would truncate the protein (146X, 199X, and 464X) and would remove the carboxy-terminal binding site to prevent SUFU’s association with GLI1. In support of this, 146X, 199X, and 464X all showed a significant loss of SUFU function. We hypothesize that the remaining missense mutation that displayed a loss of function phenotype, E376K, could cause a conformational change in SUFU to hinder its ability to bind to GLI1.

**Fig 3:**
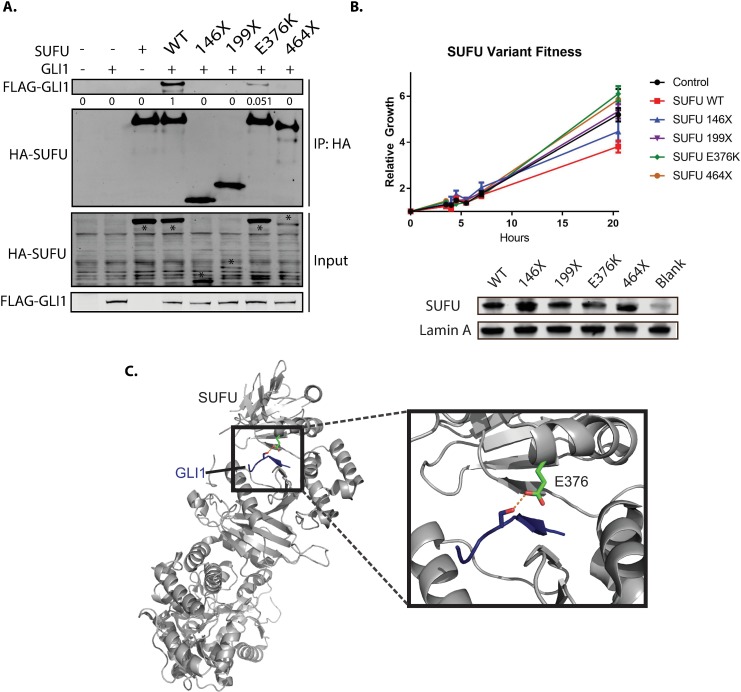
Select SUFU variants disrupt binding to GLI1. (A). HEK-293T cells were co-transfected with SUFU:TAP and GFP:GLI and co-immunoprecipitated to determine interaction between the two proteins. All of the truncation mutations (146X, 199X, and 464X) fully abolished any interaction to GLI1, while one point mutation (E376K) disrupted 94.9% of SUFU’s binding to GLI1, as shown by the quantification below the GLI1 blot. Asterisks indicate the correct SUFU band on the input blot. (B). Comparative growth assay in ASZ001 cells nucleofected with SUFU WT and SUFU variants. (C). E376K visualized on the known GLI-SUFU crystal structure, revealing that E376 forms a hydrogen bond to GLI1 in the pocket between SUFU’s lobes. Blue is GLI1, green is E376K, and the dashed red line is the hydrogen bond.

To check whether 146X, 199X, E376K, and 464X were still able to bind to GLI1, we cloned GFP-GLI1 and SUFU-TAP versions of the constructs and tested their ability to bind by co-immunoprecipitation. We co-transfected GLI1 and wild type or mutated versions of SUFU-TAP into human embryonic kidney 293T cells, immunoprecipitated SUFU, and probed for both SUFU and GLI1 protein. All 4 of the SUFU variants that disrupted normal activity were unable to suppress the Hh pathway. E376K showed 94.9% diminished binding to GLI1 and every truncation showed complete loss of interaction with GLI1 (Fig 3A), suggesting that they are no longer able to sequester and degrade GLI proteins. Co-immunoprecipitations of SUFU and GLI1 in the suspected passenger mutations from Fig 2B were also performed, and confirmed that these mutations conferred the same protein functionality to these variants as wild type (S1 Fig).

To confirm the functional effects of the SUFU mutations, we overexpressed wild type and mutated SUFU into the ASZ001 BCC cell line and observed growth changes using a real time glow luciferase assay (Fig 3B). As expected, SUFU WT suppressed the growth of the ASZ001 cells compared to control nucleofected cells after 20 hours, consistent with its role as a tumor suppressor. Each loss of function variant, however, did not significantly suppress the growth of ASZ001 cells, confirming that SUFU requires the ability to bind and inhibit GLI in order to suppress tumor cell growth.

To confirm that the loss of SUFU’s GLI1-binding activity impacts GLI1 sub-cellular localization, nuclear GLI1 depletion assays were performed. Consistent with previous reports, nuclei of NIH 3T3 cells overexpressing WT SUFU were severely depleted of GLI1 protein despite pathway activation [13]. In contrast, SUFU variants 146X, 199X, E376K, and 464X had no effect on the nuclear localization of GLI1 (S2 Fig).

### E376K Reveals an Important Interface Necessary for Proper Protein Function

It is interesting that E376K is able to dramatically disrupt SUFU’s function similar to truncated variants. In order to determine how a single point mutation could have such a dramatic effect, we examined the location and interactions of E376K in the crystal structure of SUFU and GLI1 [14]. Interestingly, E376K is at the surface between SUFU’s 2 main lobes, and normally forms a hydrogen bond with GLI1 in the binding pocket of SUFU [12]. This placement suggests E376K could help stabilize the SUFU and GLI1 interaction, as E376K appears in the crystal structure as a key side chain interaction (Fig 3C). Since E376K appeared in two independent early-stage BCCs, our data suggests we may be able to predict which SUFU variants would promote tumor growth through similar side chain interactions.

## Discussion

Our results show that clinically observed mutations in SUFU have the potential to drive tumor growth and further elucidates SUFU’s role in binding to and suppressing GLI function. Previous studies have determined SUFU’s role in sequestering GLI1 in the cytoplasm, facilitating GLI1 protein degradation, and serving as a scaffold for transcriptional co-repression with other factors including the SAP18/Sin3/HDAC1 complex (12). However, the precise molecular interaction with GLI1 has been poorly defined. Our work continues to delineate the interaction between SUFU and GLI1, and specifies which residues are important in the pathogenesis of tumor growth.

Previous structural studies with SUFU and GLI1 show that SUFU amino and carboxyl lobes form a sandwich that allows binding and inhibition of GLI1 through GLI1 residues 120–125. SUFU amino terminal residues 147–160 and carboxyl terminal residues 265–270 form the main contacts that interact with GLI1. This and other functional studies of SUFU provide the basis to predict which mutations will disrupt SUFU’s function. Consistent with this notion mutations at Y147, D159, and H164 have been previously shown to disrupt HH signaling and GLI1 stability, and would be prime candidates to promote tumor growth (10). Interestingly, our demonstration that substitution of G158 with a charged arginine had no effect on the GLI1 interaction illustrates the flexibility within the amino terminal interface for substitution. By contrast there has been limited functional validation of SUFU carboxyl terminal interactions with GLI1, and our discovery of the SUFU E376K variant defines the extent of the interface between the two lobes that regulate GLI association and suppression. Additional mutations within the carboxyl terminal side chain lobe including residues 376–380 would be expected to inhibit SUFU function.

Overall, this work adds to our understanding of the structure and function of SUFU and GLI, expands our knowledge of BCC mutations, and reinforces the idea that highly mutated cancers contain many passenger mutations of no known functional relevance. As this wealth of knowledge continues to grow, an increasing opportunity emerges for genetic pre-screening to determine the optimal personalized treatment for patients with HH-dependent cancers.

## Supporting Information

S1 Fig SUFU variants that do not increase *GLI1* transcript levels have normal protein function. HEK-293T cells were co-transfected with SUFU:TAP and GFP:GLI and co-immunoprecipitated to determine interaction between the two proteins. This figure depicts the results for the SUFU variants that showed no change in function at the mRNA level.
(EPS)

S2 Fig Nuclear/cytoplasmic fractionation assay confirms SUFU variants’ loss of function. 3T3 cells were transfected with peGFP-C1 SUFU constructs. The cells were fractionated and probed for SUFU, GLI1, and Lamin A + C, a nuclear loading control in order to assay for nuclear depletion of GLI1 in the presence of a functional SUFU. Asterisks indicate the correct SUFU band on the GFP-SUFU blot.
(EPS)
